# Infectious Disease: Evolution, Mechanism and Global Health

**DOI:** 10.1242/dmm.052701

**Published:** 2025-10-17

**Authors:** Rachel Hackett, Judith E. Allen, Sumana Sanyal, David M. Tobin, Russell Vance

**Affiliations:** ^1^ The Company of Biologists, Bidder Building, Station Road, Cambridge CB24 9LF, UK; ^2^ Lydia Becker Institute of Immunology and Inflammation, School of Biological Sciences, Faculty of Biology, Medicine and Health, Manchester Academic Health Science Centre, University of Manchester, Manchester M13 9PT, UK; ^3^ Sir William Dunn School of Pathology, University of Oxford, South Parks Road, Oxford OX1 3RE, UK; ^4^ Department of Molecular Genetics and Microbiology, and Department of Immunology, Duke University School of Medicine, Durham, NC 27710, USA; ^5^ Howard Hughes Medical Institute, University of California, Berkeley, CA 94720, USA; ^6^ Division of Immunology and Molecular Medicine, University of California, Berkeley, CA 94720, USA; ^7^ Department of Molecular and Cell Biology, University of California, Berkeley, CA 94720, USA; ^8^ Cancer Research Laboratory, University of California, Berkeley, CA 94720, USA

**Figure:**
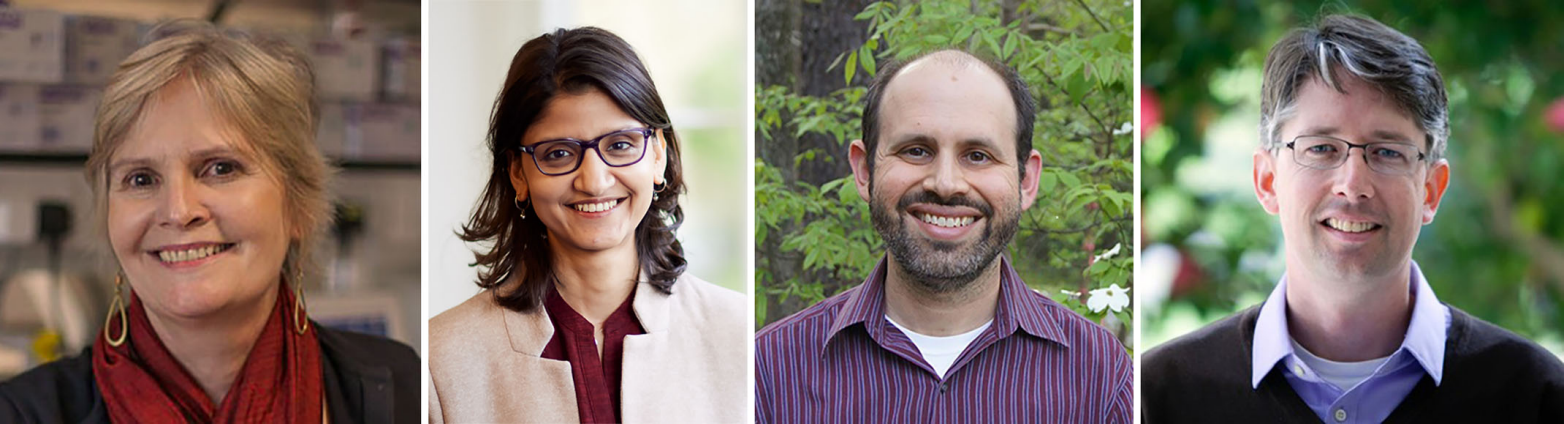
The Special Issue Editors (left to right): Judi Allen, Sumana Sanyal, David M. Tobin and Russell Vance

Infectious diseases continue to challenge clinicians, research scientists and health organisations worldwide. From early plagues to modern pandemics, there is growing appreciation of the evolving interactions between pathogen and host that lead to clinical symptoms, tolerance or resolution of infectious disease. Understanding the mechanisms by which pathogens spread, adapt and persist is crucial not only for scientific progress, but also for the development of therapies and the implementation of public health strategies.

In 2022, with COVID-19 still dominating headlines and affecting lives all over the world, long-established infectious diseases – such as HIV, tuberculosis (TB) and malaria – continuing to annually kill millions of people, and the looming global health threat of antimicrobial-resistant pathogens, there was increased emphasis on the clear and urgent need to improve our understanding of the mechanisms of pathogenesis, and to develop new strategies to prevent, diagnose and treat infection. As a journal run by scientists and embedded in the community, Disease Models & Mechanisms (DMM) was ideally positioned to support and encourage ground-breaking basic and pre-clinical research in this area (Tobin, 2022).

In October 2023, DMM hosted a meeting in London – ‘Host−Pathogen Interactions Through An Evolutionary Lens’, organised by Wendy Barclay (Imperial College London, London, UK), Sara Cherry (University of Pennsylvania, Philadelphia, PA, USA), DMM Editor David Tobin (Duke University, Durham, NC, USA) and Russell Vance (University of California, Berkeley, CA, USA). By using this ‘evolutionary lens’, leading experts explored the history of human infectious disease, pathogen emergence, virulence traits and diverse host responses, culminating in discussions around clinical consequences and therapeutic opportunities.

This gathering gave us much to contemplate. Alongside supporting disease communities by hosting meetings, DMM's core aim is to publish rigorous, high-quality research. Therefore, to continue to support advances in this field, meeting organisers David Tobin and Russell Vance, speaker Judi Allen (University of Manchester) and DMM Associate Editor Sumana Sanyal (University of Oxford) agreed to guest edit a special issue of DMM focused on Infectious Disease: Evolution, Mechanism and Global Health. The goal in shaping this special issue was to compile original Research, Resources & Methods and Review-type articles that increase our understanding of infectious disease.

One important theme that has emerged is that insights come from deep analysis of genome evolution as host and pathogens co-evolve. In our A Model for Life interview, Harmit Malik (Malik, 2025) describes how he and his colleagues made pioneering contributions to our understanding of the ‘molecular arms races’ between the viruses changing to evade host immune systems and the host antiviral proteins adapting to keep up. As Harmit says, “We cannot simply rely on the idea that the therapies currently in clinical trials are going to be enough […] there needs to be a constant influx of new ideas to stay ahead of the arms race”.

These principles span the evolution of diverse micro-organisms that cause infectious disease. Fungal infections are underappreciated as a global health threat, but fungal species such as *Candida auris* have emerged as new threats to human health; many fungal species are particularly challenging to treat. Climate change is also likely to make dangerous fungal infections more prominent in our future.

## Understanding fungal infections in a warming world

Two closely related fungi – *Cryptococcus deneoformans* (Cd) and *Cryptococcus neoformans* (Cn) – can cause serious infections. Cd is more often found in cooler climates and tends to infect the skin, whereas Cn is more widespread and more likely to affect the brain and lungs. Research by Martinez and colleagues (Charles-Niño et al., 2025) explored how Cd and Cn respond to temperature, and how they interact with human tissues, including their route of infection. Researchers found that Cd is less able to tolerate high temperatures than Cn. Cd forms sticky layers called biofilms more easily, especially on skin, which may help it to survive and hide from the immune system. By contrast, Cn is better adapted to the warmer temperatures inside the human body, partly due to higher levels of protective heat-shock proteins. These differences help explain why Cd and Cn cause different types of infections and support the idea that they should be treated as distinct species. Understanding how fungi adapt to temperature and form biofilms could inform the diagnosis and treatment of fungal infections – especially given that climate change could make these infections more common.

## Neuroinflammation in fungal infections: a double-edged sword

Fungal infections of the central nervous system (CNS) are increasingly recognised as a serious threat, particularly among immunocompromised individuals, in whom they are associated with significant neurological damage and high mortality rates. Despite their clinical severity, these infections remain under-represented in research and policy discussions. A hallmark of CNS fungal infections is neuroinflammation, but the mechanisms by which it drives pathology in fungal infections are still poorly understood, posing a major challenge to effective treatment. In their Review, Dangarembizi and colleagues explore the protective immune barriers of the CNS, the evasive strategies employed by fungal pathogens and the multifaceted nature of neuroinflammatory responses during infection (Dangarembizi et al., 2025). By examining current knowledge and identifying gaps in understanding, the Review calls for renewed attention to this neglected field. Advancing research into the immunopathogenesis of CNS fungal infections is essential for developing targeted therapies and improving outcomes for vulnerable patient populations.

## New ways to study lung infections caused by emerging non-tuberculous mycobacteria

Similarly to emerging fungal pathogens, non-tuberculous mycobacteria (NTM) comprise a group of bacteria found in soil and water that have also seen remarkable growth as human pathogens in the past decades. They can cause several types of infections, including skin and soft tissue infections and, most frequently, pulmonary disease. Such infections are becoming more common, and treatment of NTM lung disease can be complex because of the intrinsic antibiotic resistance of NTM, medication side effects and extended duration of drug treatment. More efforts are needed to develop new, safe and efficacious therapeutics, and to shorten treatment duration.

*Mycobacterium abscessus* is a rapidly emerging NTM that causes chronic lung disease. To help researchers develop treatments, Lamichhane and co-workers (Rimal et al., 2025) created a cost-effective mouse model using a common laboratory strain (BALB/c). As these mice naturally clear the infection, they were given a mild immunosuppressant to allow the bacteria to grow and mimic chronic disease. This method successfully reproduced the infection pattern seen in humans, and the model was then used to test antibiotics. One drug, imipenem, significantly reduced the bacterial load, whereas another, clofazimine, only slowed bacterial growth. This accessible mouse model makes it easier for laboratories around the world to study *M. abscessus* and test new treatments. It's a valuable step toward finding more effective therapies for a difficult-to-treat lung disease.

In addition, new technologies are increasing our knowledge of mycobacterial interactions with human hosts. Shiloh and colleagues developed a humanised, three-dimensional, alveolus lung-on-a-chip (ALoC) model of *Mycobacterium fortuitum* lung infection (Ektnitphong et al., 2025). Developing *in vitro* models that can recapitulate the human lung microenvironment using primary human cells has the potential to advance the study of pulmonary NTM infections. The AloC model is an innovative microfluid device composed of microfabricated channels and chambers that mimic the architecture and cellular composition of the lung alveolus. When *M. fortuitum* was introduced into the chip, the infection triggered strong immune responses, including the release of signalling molecules and protective proteins. This new model could help scientists study how lung infections start and how the body responds – and could also be used to test new antibiotics and better understand other types of NTM infections or TB.

To fully understand the interactions between host and pathogen, it will be crucial to have detailed knowledge of the cell biology of how pathogens – viruses, bacteria, fungi and parasites – interact with host cells, invade tissues and manipulate cellular processes. These interactions are central to disease progression and immune response; several articles explore these aspects.

## Neutrophil time bombs: how immune cells keep their options open during infection

Neutrophils are vital to the immune system's early defence against multiple pathogenic microbes, but can also play pathogenic roles within the host in some infections. Analyses of zebrafish and human neutrophils conducted by Muir, Condliffe, Renshaw and co-workers surprisingly showed that a subset of neutrophil phagosomes can remain unsealed (Muir et al., 2025). Therefore, neutrophils might retain the ability to modulate phagosome content post-engulfment, potentially influencing microbial killing, antigen presentation and intercellular signalling. This discovery opens new avenues for understanding neutrophil plasticity and immune regulation during infection, and could help explain why some immune responses are stronger or weaker than expected.

## Disrupted trafficking: a TOM1 variant drives autophagy failure and autoimmunity

The G307D variant of TOM1, an endosomal adaptor protein, has been linked to inborn errors of immunity – conditions that manifest as increased susceptibility to infections, autoimmunity, autoinflammation, severe allergy and malignancy. Ryhänen et al. used patient cells to show that this mutation disrupts the interaction of TOM1 with TOLLIP, impairing both cargo trafficking and autophagy regulation (Lång et al., 2025). Their data highlight the importance of such fundamental cellular functions in controlling immune responses. Their study also provides insights into the drawbacks of immunomodulatory and stem cell therapies for patients with TOM1 pathogenic variants, and may also inform our understanding of other inborn errors that similarly affect the regulation of innate immunity.

## Can fighting a virus harm the brain?

Intracerebral haemorrhage (ICH) is a type of stroke caused by the rupture of brain blood vessels and subsequent bleeding within the brain. In rare cases, infection can lead to such rupture. Although the mechanism by which this occurs is poorly understood, the antiviral enzyme cholesterol 25-hydroxylase (CH25H) and its metabolite 25-hydroxycholesterol (25HC), which modulates cholesterol metabolism during infection, have been implicated. Using a zebrafish model and human brain tissue, Kasher and colleagues found that high levels of CH25H and 25HC were linked to small brain bleeds following COVID-19 infection (Tapia et al., 2025). They showed that 25HC can damage endothelial cells – especially when cholesterol levels are low; when cholesterol was added, damage was reduced. This work highlights the importance of cholesterol homeostasis in maintaining the function of blood vessels in the brain and suggests that it could be a key link between viral infection and ICH. Given the health issues caused by COVID-19 and our limited understanding of long-term complications, it is important to improve our mechanistic understanding of these processes to guide future preventative strategies or treatments.

## How the immune system fights tuberculosis – and what happens when it fails

Our immune system uses special structures called inflammasomes to detect and respond to harmful bacteria, including those that cause TB. Rämet and colleagues used zebrafish to explore the role of a key inflammasome adaptor protein called ASC/PYCARD (Uusi-Mäkelä et al., 2025). By creating zebrafish with mutations in the *pycard* gene, scientists found that, although young fish could still fight off *Mycobacterium marinum* infection, which mimics TB, adult fish were more vulnerable and showed impaired survival and higher bacterial burden. Transcriptome analysis with RNA sequencing of zebrafish haematopoietic tissue suggested a role for *pycard* in neutrophil-mediated defence, haematopoiesis and myelopoiesis during infection. This research highlights the importance of ASC in mycobacterial pathogenesis *in vivo* and shows how different parts of the immune system work together.

## Seeing TB in 3D

In their Resources & Methods article, Beltran and colleagues describe a novel 3D imaging approach that visualises how the lung granulomas that are characteristic of TB infection form, evolve and spatially organise (Beltran et al., 2025). Their approach combines passive CLARITY (PACT)-based clearing with light-sheet fluorescence microscopy to visualise lesion architecture in infected mice. They also outline a method for volumetric correlative light and electron microscopy, enabling tracking of individual immune cell populations within granulomas. Overall, this provides a comprehensive and unbiased method for investigating TB infection in 3D, further highlighting the importance of considering the 3D spatial relationships within lung tissue to better understand the pathophysiology of TB and optimise treatment approaches.

In our fascinating The Patient's Voice interview, DMM talked to Dr Zolelwa Sifumba – a clinician, researcher and global health activist (Sifumba, 2025). She is a survivor of multi-drug resistant TB, which she contracted through occupational exposure while working as a medical student in South Africa. She highlights how this experience, along with her training as a medical doctor, complements her work as an activist, and how the scientific and medical communities should positively push towards patient empowerment by directly involving patients in cutting-edge TB research. Indeed, patient advocacy can inform research in TB and other infectious diseases (both acute and chronic) in unique ways that can massively improve the pace and quality of diagnoses, treatment and preventative care.

## Gut immunity holds the key to a rare COVID-19-linked illness in children

Multisystem inflammatory syndrome in children (MIS-C) is a rare but serious condition that can occur after COVID-19 infection. It causes widespread inflammation and problems with immune cells, specifically γδ T cells, which help fight infection. Interestingly, MIS-C shares features with Kawasaki disease and inflammatory bowel disease, despite their being clinically distinct, suggesting that problems with gut immunity are a common feature. Sancho-Shimizu and colleagues (Santillo et al., 2025) give their perspective on the common link between these diseases **–** the potentially crucial role gut immunity plays in the initiation and persistence of disease through the tight regulation of γδ T cells. Understanding this link could aid prevention and treatment of these conditions.

## COVID-19 affects more than just the lungs – and we need better models to study it

Hundreds of millions of people have been infected and more than 7 million have died since the start of the COVID-19 pandemic (data from World Health Organization). Although the COVID-19 virus is known to cause lung problems, many patients also experience symptoms such as chest pain, stroke, loss of smell and taste, diarrhoea and abdominal pain. In some people, these symptoms continue long after the initial infection, a condition known as long COVID or post-acute sequelae of COVID-19. However, the molecular mechanisms underlying the acute and systemic conditions associated with COVID-19 remain incompletely defined. Amos-Landgraf and colleagues review current research on the mechanisms of the cardiovascular, neurological and gastrointestinal pathobiology caused by COVID-19, and describe the established animal models that can be used for their study (Chung et al., 2025). By choosing the right models, researchers can more accurately explore how the disease progresses and test new treatments.

## Bats can carry deadly viruses without getting sick

Bats are natural hosts for many dangerous viruses – including those that cause diseases such as Ebola, severe acute respiratory syndrome (SARS) and COVID-19 – but they rarely get ill themselves. This is because bats have evolved a unique immune system over millions of years that allows them to live in balance with viruses.

Since the COVID-19 pandemic, bats have gained attention as a likely source of the virus's ancestor. Ng and Wang describe the usage of advanced ’omics technologies to study the mechanisms of bat immunity in detail. They give a timely and fascinating perspective on the possible translation of discoveries from bats to humans, in the context of treating and preventing infectious disease (Ng and Wang, 2025).

## Engineered bacteriophages could help fight antibiotic resistance

A 2014 report on antimicrobial resistance (AMR) warned that drug-resistant infections could lead to as many as 10 million deaths by 2050. Although the accuracy of this number has since been debated, it highlights AMR as potentially the largest single threat to human health in many of our lifetimes (GBD 2021 Antimicrobial Resistance Collaborators, 2024). The COVID-19 pandemic taught us that new antimicrobial agents are needed against emerging pathogens, but the drugs we have used to combat existing pathogens for many decades have become increasingly less effective. One promising solution is phage therapy – treatment of pathogenic bacterial infections using bacteriophages (viruses that infect bacteria). Phages are highly targeted, cause minimal side effects and can multiply at the site of infection. In their comprehensive ‘At a Glance’ poster article, Mihajlovski, Sagona and colleagues highlight how engineered bacteriophages can be tailored to enhance antibacterial therapies and diagnostics, and to support innovative biomedical applications beyond AMR challenges, potentially transforming how we manage bacterial diseases (Traore et al., 2025).

## Conclusions

The articles in this special issue highlight the breadth of research encompassed by the infectious disease research community. By investigating these individual disorders using differing and complementary approaches and models, common themes can emerge. International and cross-disciplinary collaboration and clinical partnership are also essential for the progression of infectious disease research. In addition, the climate crisis is expected to expand the geographical range of diseases, meaning that environmental and clinical strategies for their control will have to be adapted. By building a research toolkit, we are better prepared to circumvent future infectious disease threats (Sanyal, 2023). We look forward to future research advances that will undoubtedly enrich DMM's ongoing subject collection and the field as a whole.
